# Expression of chemokine CXCL8/9/10/11/13 and its prognostic significance in head and neck cancer

**DOI:** 10.1097/MD.0000000000029378

**Published:** 2022-07-29

**Authors:** Zhenyu Zhao, Yuyu Ma, Jie Lv, Naifeisha Maimaiti, Jingyi Zhang, Madinaimu Aibibula, Zhongcheng Gong, Bin Ling

**Affiliations:** a School of Stomatology, Jilin University, Changchun, Jilin, P.R. China; b State Key Laboratory of Pathogenesis, Prevention and Treatment of High Incidence Diseases in Central Asia, Clinical Laboratory Center, Tumor Hospital Affiliated to Xinjiang Medical University, Urumqi, Xinjiang, P.R. China; c Department of Oral Maxillofacial Oncology Surgery, The First Affiliated Hospital of Xinjiang Medical University, Stomatology School of Xinjiang Medical University, Stomatology Research Institute of Xinjiang Uyghur Autonomous Region, Xinjiang, P.R. China.

**Keywords:** bioinformatics analysis, biomarkers, CXCL8/9/10/11/ 13, head and neck squamous cell carcinoma

## Abstract

**Background:**

Head and neck cancer (HNC) is a very popular cancer, with many primary sites and pathological types, at the top of the list of tumors. Chemokines are a class of small molecular basic proteins, whose N-terminal cysteine residues can be divided into four subunits by location and number, which significantly enhances the expression level in all kinds of cancers. However, in HNC, especially in head and neck squamous cell carcinoma, the chemokine CXCL8/9/10/11/13 has not been clearly explored for its diagnosis and prognosis.

**Methods:**

The ONCOMINE database was used to analyze the expression of chemokine family in various cancers. After CXCL8/9/10/11/13 was screened out, the expression of CXCL 8/9/11/13 in patients with HNC/normal people were analyzed by UALCAN database. The expression and pathological stages of CXCL 8/9/10/13 in HNC tissues were analyzed by the GEPIA database, and the relationship between its mRNA expression and the overall survival (OS) time of patients with HNC was analyzed by Kaplan–Meier plotter database. In addition, 171 co-expressed genes significantly related to CXCL8/9/10/11/13 mutation were screened by online tool cBioPortal, and the protein interaction network of these genes was constructed by STRING database. Finally, the potential functions of CXCL8/9/10/11/13 and its 171 co-expressed genes were explored by the enrichment and analysis function of David database.

**Results:**

Transcriptional expression of chemokine 8/9/10/11/13 was significantly increased in patients with HNC. Clinical stage of patients with HNC was significantly correlated with overexpression of CXCL9/10/11. In addition, the chemokine CXCL8/9/10/13 was significantly correlated with over-survival of patients with HNC, so it could be distinguished between short-term and long-term survival of patients with HNC. In conclusion, CXCL8/9/10/11/13 closely connected with the expression and prognosis of HNC.

**Conclusion:**

In this study, our results suggest that chemokine CXCL8/9/10/11/13 may play a critical role in the development of HNC, and, according to relevant data, it may affect the survival and prognosis of patients with HNC.

## 1. Introduction

Mortality from head and neck cancer (HNC) has not significantly improved in recent decades. However, the molecular characteristics of HNC have not been explored. Highly specific and sensitive biomarkers can not only help to elucidate the molecular mechanism of HNC patients, but also improve the prognosis of HNC patients.^[1]^ Head and neck squamous cell carcinoma (HNSCC), which includes cancers of the oral cavity, oropharynx, and larynx/hypopharynx, is the sixth most incident cancer worldwide, with an estimated 700,000 new cases in 2018, and portends a grave prognosis with 350,000 of these predicted to be fatal.^[2]^ It is the sixth most frequent cancer in the world and this disease is recognized in half a million patients every year.^[3]^

Chemokines or chemotactic cytokines besides their functions in the immune system, they also play a critical role in tumor initiation, promotion and progression.^[4]^ Moreover, a study verified that the overexpression of C–X–C chemokine receptor 4 (CXCR4) and CXCR7 in primary head and neck squamous carcinoma (HNSC) was related to lymph node metastasis. A comprehensive study of the CXC family of chemokines in HNC will help to uncover the molecular mechanisms involved in the development of HNC could unveil novel prognostic and therapeutic targets for the intractable disease.^[5]^ CXCL8 is a chemokine as modulate tumor proliferation, invasion and migration in an autocrine or paracrine manner. Studies have suggested that CXCL8 and its cognate receptors, CXCR1 and CXCR2, mediated the initiation and development of various cancers.^[6]^ Relevant studies have shown that the paracrine axis of CXCL9/10/11, CXCR3 regulated immune cell migration, differentiation and activation, leading to tumor inhibition, while the autocrine axis was involved in tumor growth and metastasis.^[7]^ The expression of chemokine CXCL8/9/10/11/13 in a variety of cancers can be better understood from the Previous study, and even promote the growth and development of tumors. In the existing study, the role of chemokine CXCL8/9/10/11/13 in HNC is not elaborated. This study demonstrated the relevant biological information of CXCL8/9/10/11/13 in the expression and prognosis of patients with HNC.

## 2. Materials and Methods

### 2.1. Ethics statement

The information of the data is from the online database, so the data was obtained in accordance with the written informed consent.

### 2.2. ONCOMINE database

ONCOMINE database (Note 1) is a publicly accessible online cancer database, which provides a genome-wide expression analysis.^[8]^ Oncomine database was used to analyze the difference in transcription levels between different types of cancer tissues/normal tissues next to cancers. When using the database, the *P*-value is .05, the folding change value is 2, and the gene ranks the top 10%.

### 2.3. UALCAN

UALCAN (Note 2) is based on The Cancer Genome Atlas (TCGA) level 3 RNA-seq and clinical data from 31 cancer types.^[5]^ In this study, the UALCAN database was utilized for exploring different types of expression between HNC tissues/normal tissues. *P* ≤ .05 is statistically meaningful.

### 2.4. GEPIA

GEPIA (Note 3) is expounding RNA sequencing and expression data from the cancer genome atlas (TCGA) and genotype tissue expression data sets in 9736 tumors and 8587 normal samples. GEPIA provides functions such as tumor/normal differential expression analysis, analysis according to cancer type or pathological stage, patient survival analysis, similar gene detection, correlation analysis.^[9]^ In this study, the polygene comparative analysis function of GEPIA database was used to conduct the relative polygene comparative analysis of the chemokine CXCL family, and the relationship between CXCL8/9/10/11/13 and clinical stage pathological parameters was explored respectively by the expression DIY function. *P* ≤ .05 is statistically meaningful.

### 2.5. Kaplan–meier plotter

The Kaplan Meier plotter (Note 4) is able to assess the effect of 54,000 genes on survival in 21 cancer types.^[10]^ In this study, the GEPIA database was used to evaluate the prognostic value of chemokines, such as CXCL8/9/10/11/13 in HNC, and its correlation was verified by the K–M survival curve, and its risk ratio (HR) was based on the confidence interval and *P*-value of 95%. When *P* ≤ .05 is statistically meaningful.

### 2.6. cBioPortal

CBioportal database epigenetic cancer tissue, gene expression and gene mutation provide graphical results.^[11]^ In this article, we used cBioportal database to explore the gene profiles of CXCL8/9/10/11/13, and their gene mutations (Fig. 6). For patients with HNC, both over survival (OS) and disease-free survival (DFS) was analyzed by Kaplan–Meyer. When *P* ≤ .05 is statistically meaningful. We also explored the association between the chemokine CXCL8/9/10/11/13 by co-expression analysis of cBioportal.

**Figure 1.**
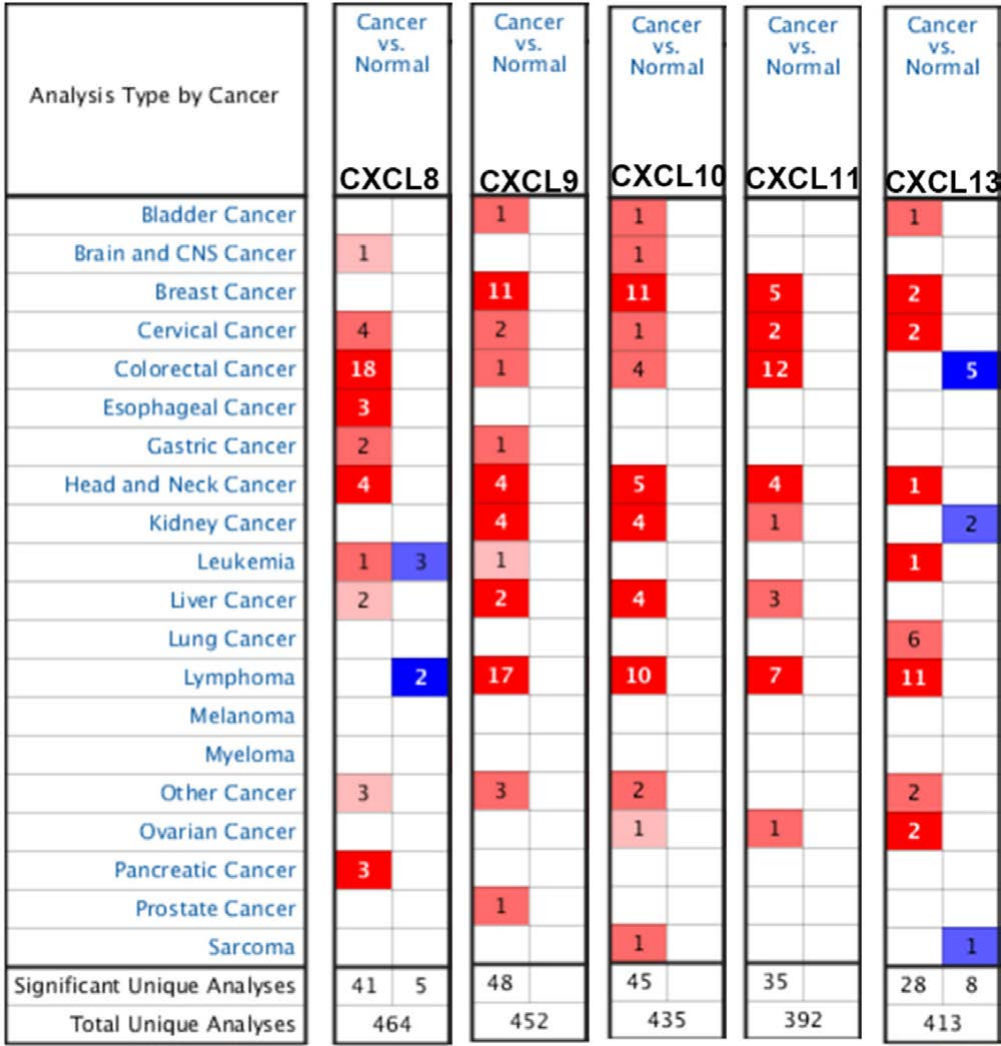
The mRNA expression of the CXCL8/9/10/11/13 in all kinds of cancer was red when the expression was positive adjustment and blue when the expression was reverse adjustment. This figure was collected from a data set with relevant statistical significance. *P*-value and the fold change value are shown as follows: *P*: .01, fold change: 2, gene grade: 10%, data type: mRNA.

**Figure 2.**
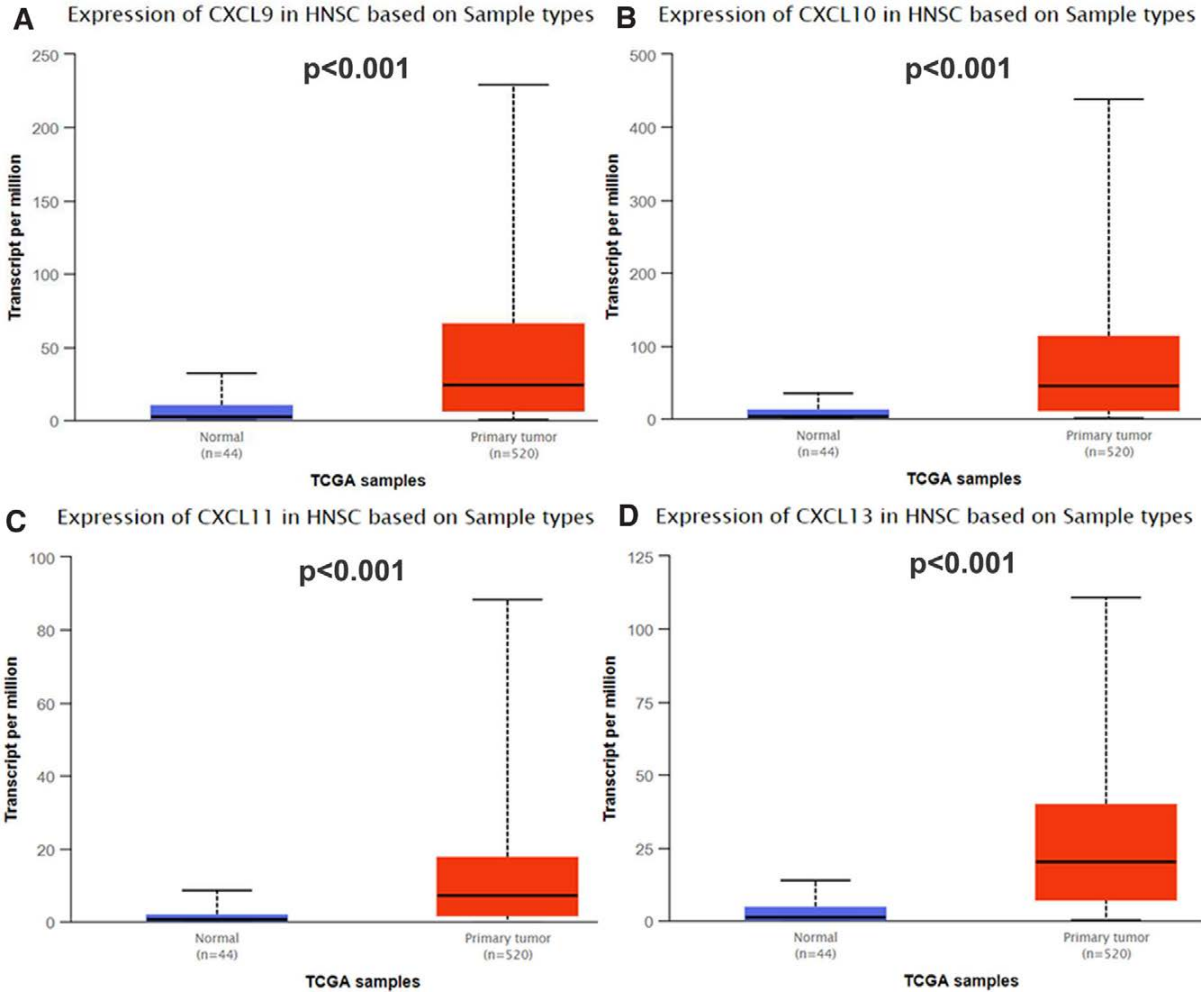
The expression of CXCL8/910/11/13 in the diseased tissues of patients with HNC compared with adjacent normal organizations, and the box chart exhibited that the mRNA expression of the chemokine CXCL9/10/11/13 was statistically significant.

**Figure 3.**
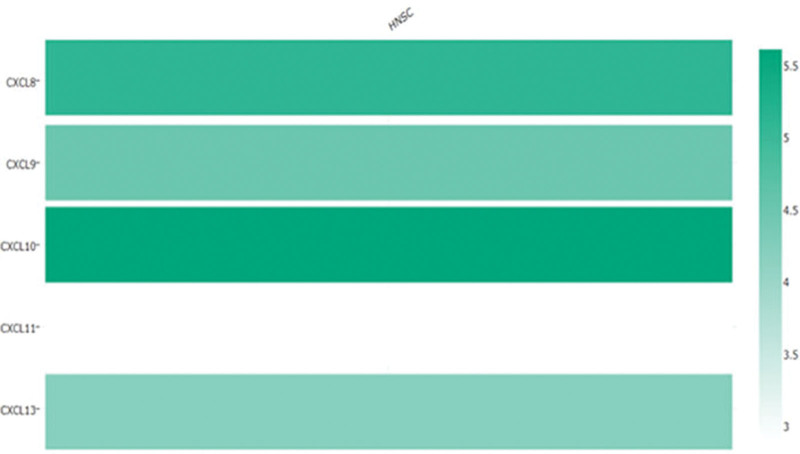
Relative expression level of CXCL8/910/11/13 in HNC cancer. The higher the expression of mRNA, the darker the color in this figuer, and the higher the expression level of CXCL10 in HNC cancer was found.

**Figure 4.**
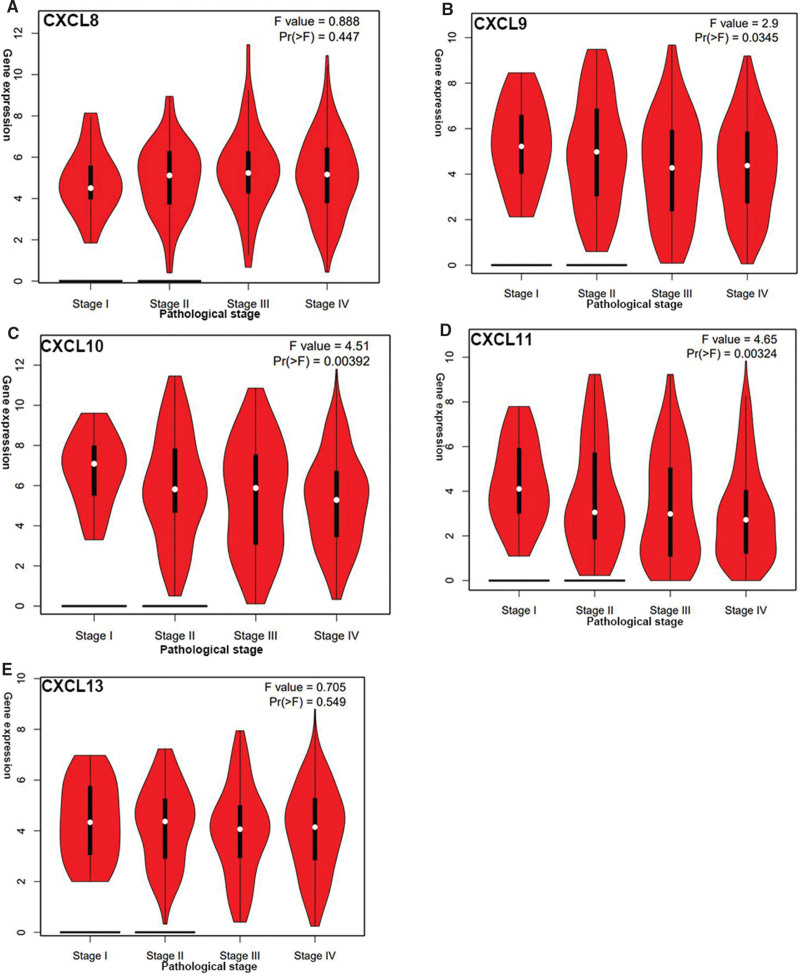
The expression of CXCL8/9/10/11/13 is related to tumor staging. The expression of mRNA in CXCL9/10/11 is obviously connected with the stage of cancer in patients, while the expression of mRNA in CXCL8/13 is not about it.

**Figure 5.**
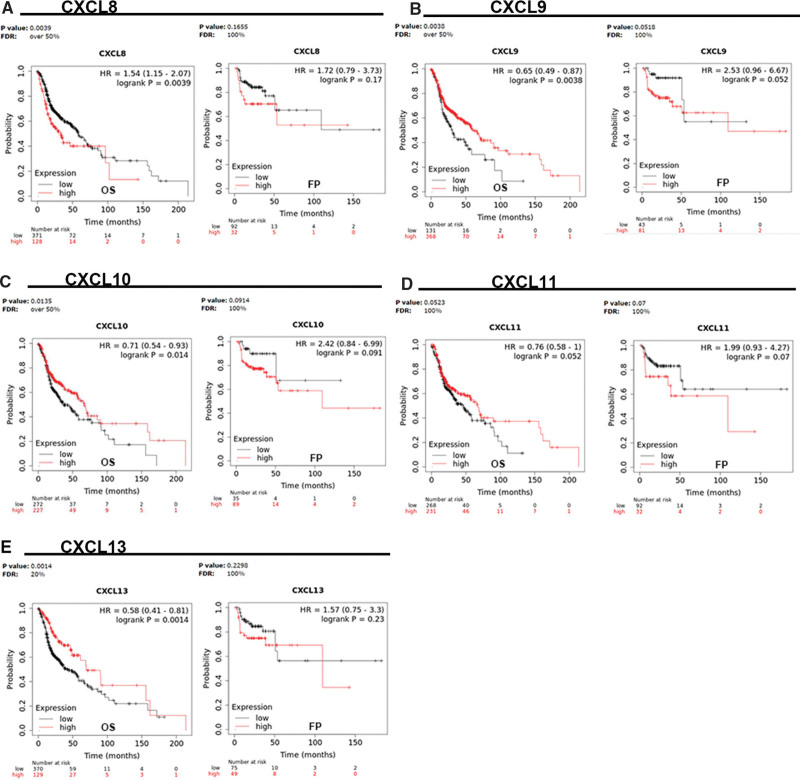
We use Kaplan–Meier plotter database (Note 4) to explore the connection of CXCL8/9/10/11/13 and Over survival (OS), and draw the relevant survival curve. Red is high expression, while black is low expression. The relationship between CXCL 8/9/10/13 and OS is statistically significant. The *P*-values are .0039, .0038, .014, and .0014 individually. The *P*_(CXCL8/9/10/11/13)_ of FP was > .05, which had no statistical significance.

**Table 1:** Remarkable changes of the CXCL8/9/10/11/13 expression in transcription level between HNC/normal tissues (ONCOMINE).

	Type of HNC	Fold change	*P*	*t*-Test
CXCL8	Tongue Squamous Cell Carcinoma vs Normal	10.158	7.11E-11	9.644
	Head and Neck Squamous Cell Carcinoma vs Normal	36.405	2.12E-15	13.407
	Oral Cavity Squamous Cell Carcinoma vs Normal	20.342	2.76E-20	14.056
CXCL9	Oral Cavity Squamous Cell Carcinoma vs Normal	16.067	1.51E-18	12.430
CXCL10	Nasopharyngeal Carcinoma vs Normal	7.729	7.60E-12	11.631
	Head and Neck Squamous Cell Carcinoma vs Normal	6.410	1.15E-11	8.488
	Oral Cavity Squamous Cell Carcinoma vs Normal	19.340	6.43E-16	12.378
CXCL11	Nasopharyngeal Carcinoma vs Normal	14.625	5.58E-10	10.466
	Oral Cavity Squamous Cell Carcinoma vs Normal	20.050	4.64E-17	11.661
CXCL13	Head and Neck Squamous Cell Carcinoma vs Normal	67.011	3.47E-16	14.825

### 2.7. STRING

STRING6 (Note 6) is a database of protein–protein interactions (PPI) interactions.^[12]^ For the sake of exploring the role of the co-expression gene of chemokine CXCL8/9/10/11/13, we used the STRING database to analyze the relationship between the chemokine CXCL8/9/10/11/13 and the PPI network. According to relevant requirements, choosing Homo sapiens as species types and combining scores > 0.7 are considered statistically significant in clinical practice. The nodes in the figure represent proteins.

### 2.8. DAVID

Mutational function of Chemokine CXCL8/9/10/11/13 and 171 genes was analyzed by Gene ontology (GO) and Kyoto encyclopedia of genes and genomes (KEGG) in the DAVID database.^[13]^ Ontology analysis of genes focuses on three major areas, such as biological processes (BP), cellular components (CC), and molecular functions Molecular functions (MF). Correlation analysis was significant in predicting mutations of CXCL8/9/10/11/13 and 171 genes. When *P* ≤ .05 is statistically meaningful.

## 3. Results

### 3.1. Aberrant expression of chemokines CXCL8/9/10/11/13 in patients With HNC

In Oncomine database and UAICAN database, we analyzed the expression of CXCL8/9/10/11/13 in HNC tumors to find the difference in the expression level of CXCL8/9/10/11/13 in tumor and normal samples (Fig. 2). The results exhibited in Figure 1 showed that mRNA expression levels of CXCL8/9/10/11/13 differed significantly between HNC tissues / normal tissues in a large number of databases (Fig. 2). The result of Figure 1 also displayed that the mRNA expression of CXCL8/9/10/11 differed significantly between HNC and normal tissues. In Ye Head-Neck Statistics CXCL8 excessive expression of CXCL8 in HNC (N = 41) compared with normal tissues (N = 5) with a fold change of 10.158 (*P* ≤ .01) (Table 1), while Ginos Head-Neck Statistics found a fold change of 10.158 in mRNA expression (*P* ≤ .01) (Table 1). Moreover, Peng Head-Neck Statistics prospected a 3.9n addition 28-fold increase in CXCL8 mRNA expression in 79 HNC samples (*P* ≤ .01) (Table 1), CXCL9 rase obviously in HNC tissues compared to normal tissues. The result from the Peng Head-Neck Statistics unfolded that there was a fold change of 16.067(*P* ≤ .01) (Table 1) increase in CXCL9 mRNA expression in 79 HNC tissues. In Sengupta Head-Neck Statistics, CXCL10 was overexpressed in HNC by a fold change of 7.729 (*P* ≤ .01) (Table 1) in HNC. In the Sengupta Head-Neck Statistics, the mRNA expression of CXCL11 in patients with HNC (N = 41) rose with a fold change of 14.625 (*P* ≤ .01) (Table 1). In the Peng Head-Neck by a fold change of 20.050 (*P* ≤ .01) (Table 1). The transcriptional levels of CXCL13 in 54 in patients with HNC (fold change = 67.011, *P* ≤ .01) (Table 1). In addition, we compared the expression of the CXCL8/9/10/11/13 gene in the UALCAN database, and found that the expression of CXCL8/910/13 was more significant in patients with HNC, especially in the expression of the CXCL10. In conclusion, the gene expression of the CXCL8/910/11/13 may regard as a new genetic target for the occurrence, prediction and prognosis of on patients with HNC (Fig. 3).

### 3.2. Associations between mRNA expression levels of the chemokine CXCL8/910/11/13 and clinical stage in patients with HNC

GEPIA database was used to explore the relationship with mRNA expression of CXCL8/9/10/11/13 and cancer stage of patients with HNC. As shown in Figure 4, the *P*_(CXCL9/10/11)_ ≤ .01. However, the *P*-value of CXCL8/13 has not relevant statistical significance. It might relate to the patient specimens collected in the database were not comprehensive and complete. In conclusion, mRNA expression of CXCL9/10/11 is correlated with tumor cancer stage in patients with HNC, even CXCL9/10/11 was more expressed in patients with HNC at advanced stage.

### 3.3. CXCL8/910/11/13 characteristics have relationship with the prognosis of patients with HNC

We used the Kaplan–Meier plotter database to explore the correlation between the CXCL8/9/10/11/13 and prognosis of in patients with HNC. The relationship of CXCL8/9/10/11/13 mRNA expression with OS and FP was analyzed in this study (Table 2) (Fig. 5). *P*_(CXCL8/9/10/13)_ ≤ .01 in OS.

**Table 2:** The prognostic values of the CXCL8/9/10/11/13 in HNC patients (Kaplan–Meier plotter).

	OS				FP			
The CXCL family	Cases	HR	95% CI	*P*	Cases	HR	95% CI	*P*
CXCL8	7642	1.54	1.15–2.07	.0039	4420	1.72	0.79–3.73	.1655
CXCL9	7642	0.65	0.49–0.87	.0038	4420	2.53	0.96–6.67	.0518
CXCL10	7642	0.71	0.54–0.93	.0135	4420	2.42	0.84–6.99	.091
CXCL11	7642	0.76	0.58–1	.052	4420	1.99	0.93–4.27	.07
CXCL13	7642	0.58	0.41–0.81	.0014	4420	1.57	0.75–3.3	.23

### 3.4. The CXCL8/9/10/11/13 genetic mutations and their genetic changes take effect in patients with HNC

**Figure 6.**
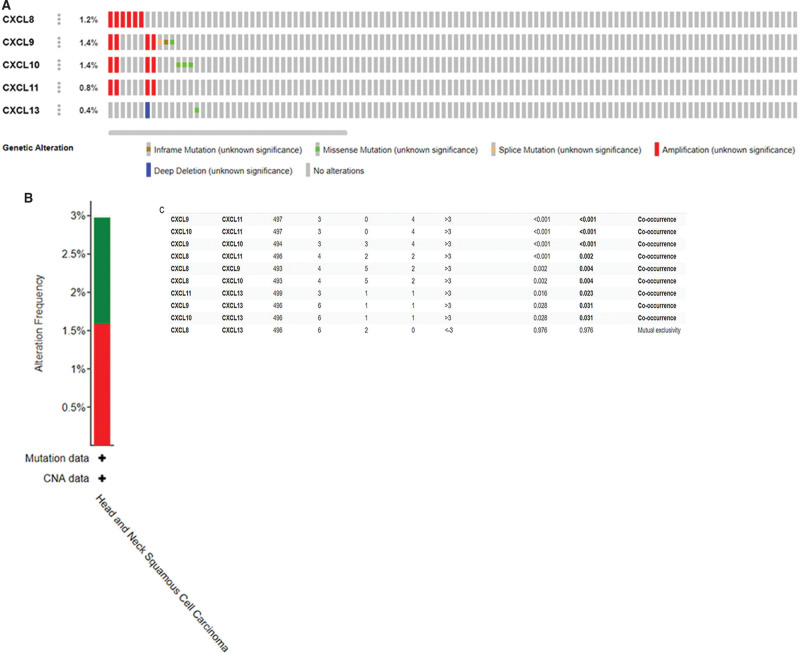
The relationship between patients with HNC and CXCL8/9/10/11/13 gene mutation (A). *P*_(CXCL8/9/10/11/13)_ ≤ .05 in Table 1 indicates that there was a positive relationship between its.

We used cBioportal online tool to analyze the mutation of CXCL8/9/10/11/13 in patients with HNC (Fig. 6A). This database showed that there was a genetic mutation in CXCL factor in 15 out of 504 patients with HNC. In which CXCL8/9/10/11 is the most representative genes of the gene mutation, with the mutation probability of 1.2%, 1.4%, 1.4%, 0.4%, and 0.8% respectively (Fig. 6). In addition, we also analyzed the correlation between CXCL8/9/10/11/13 through cBioportal online tool. The results displayed that the mutation of CXCL13 was positively correlation in this study. CXCL8/9/10/11/13 probably provide a new genetic target for the early diagnosis in patients with HNC.

### 3.5. Explore the enrichment of CXCL8/9/10/11/13 in patients with HNC

**Figure 7.**
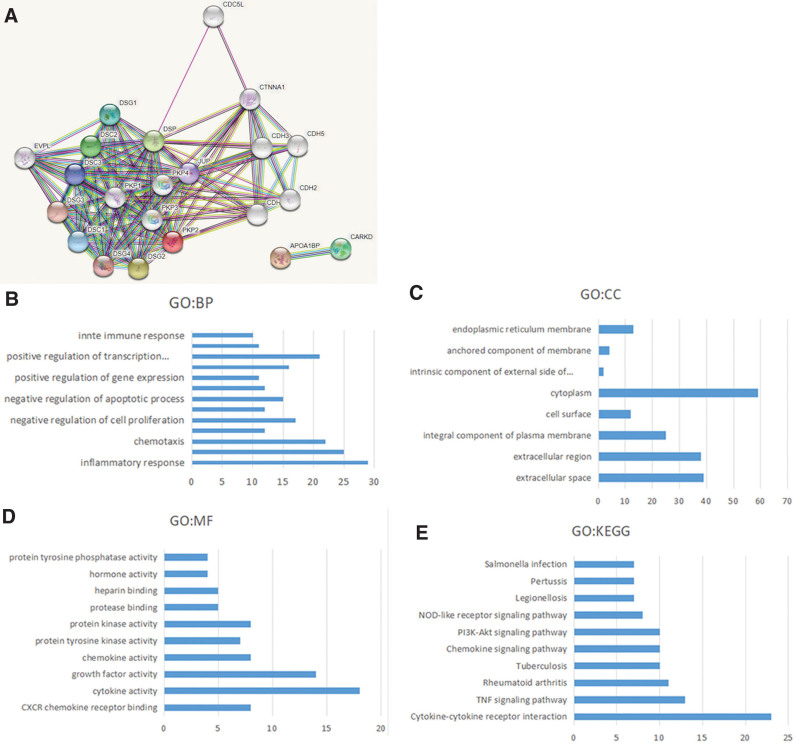
CXCL8/9/10/11/13 in patients with HNC and its GO and KEGG enrichment analysis of a total of 171 genes. These nodes mean proteins, and the edges mean the interaction between proteins (A). (B) BP. (C) CC. (D) MF. (E) KEGG. HNC = head and neck cancer, KEGG = Kyoto Encyclopedia of genes and genomes.

We used the co-expression analysis function of cBioportal online tool to enrich 171 co-expressed genes. Then, we built an integrated network (Fig. 7A) using the String online tool, which showed that DSC3, DSG3, PKP1, and PKP4, were closely related to the CXCL8/9/10/11/13. What’s more, David’s GO and KEGG enrichment functions were used to analyze the potential role of CXCL8/9/10/11/13 and their 171 co-expression genes, it was shown in Figure 7B, we discovered that BP (Biological processes) such as GO:0006954 (inflammatory response), GO:0006955 (immune response), GO:0043066 (positive regulation of cell proliferation), GO:0008285 (negative regulation of cell proliferation), and GO:0008283 (negative regulation of apoptotic process) were obviously regulated by the CXCL8/9/10/11/13 mutations in HNC. Moreover, Cellular components (CC), including GO:0005615 (extracellular space), GO:0005576 (extracellular region), GO:0005887 (integral component of plasma membrane), and GO:0005737 (cytoplasm). The cytoplasm was significantly associated CXCL8/9/10/11/13 with alterations (Fig. 7C). CXCL8/9/10/11/13 mutations also significantly affected the Molecular functions (MF), like GO:0005125 (cytokine activity), GO:0008083 (growth factor activity), GO:0045236 (CXCR chemokine receptor binding), and GO:0008009 (chemokine activity) (Fig. 7D). In the KEGG analysis, just hsa04530 (Tight junction) was noticeably related to the functions (Fig. 7E).

## 4. Discussion

Head and neck cancer (HNC) is the eighth common cancer worldwide, with more than 835,000 new cases and 431,000 deaths due to the disease per year.^[2]^ HNC comprises tumors in the oral cavity, pharynx, and larynx, nearly 95% are squamous cell carcinoma histological type tumors.^[14]^ Most HNC are already at an advanced stage when diagnosed, which significantly reduces the survival rate, even after curative treatment.^[15]^

Chemokines are a group of small molecular weight proteins that are structurally related. These molecules play an important role in the growth, differentiation and activation of many types of cells.^[12,16]^ Chemokines are synthesized mostly by leukocytes and act through their cognate G-protein coupled receptors to cause a cellular response, such as migration, adhesion and chemotaxis.^[12,17]^ The chemokine family has been classified into four classes: CC, CXC, CX3C, and (X), based on the arrangement of N-terminal cysteine residues.^[18]^ This article mainly discussed the relationship between CXCL8/9/10/11/13 and HNC, as well as the prognosis of gene mutations and related cancers.

Some studies have shown that CXCL8 directly contributes to tumor microenvironment remodeling, cancer plasticity, and development of resistance to chemotherapy and immunotherapy. Further, clinical data demonstrates that CXCL8 can be an easily measurable prognostic biomarker in patients receiving immune checkpoint inhibitors.^[19]^ This also confirmed the significance of *CXCL8* gene in predicting the prognosis of patients with HNC in our research. Mechanistic analyses determine that CXCL8 and its receptors contribute to the survival of CRC cells, mainly by reducing the survival pressure of tumor cells and helping them become resistant to apoptosis before and after invading the blood. Moreover, CXCL8 and its receptors are also involved in the mechanism of CRC cells and circulating CRC cells escaping from immune surveil-lance.^[20]^ Existence of *CXCL8* gene reduced the survival pressure of tumor cells to a certain extent, and it built a good growth microenvironment for tumor cells, which also has a great influence on the prognosis and survival of patients with HNC. From our research data, we can see that *CXCL8* gene has a significant statistical difference on the total survival time of patients with HNC. It was discovered the high expression of CXCL8 gene led to OS (*P* ≤ .0039) drops significantly of patients with HNC.

The prognosis of CXCL13 in different cancers is controversial. Huang et al discovered that CXCL13 highly expressed in cutaneous melanoma than normal tissue and associated with better overall survival.^[21]^ Ignacio et al showed that CXCL13 was related to longer OS in ovarian cancer with wild-type TP53.^[22]^ In contrast, Zheng et al demonstrated that CXCL13 was significantly upregulated in clear cell renal cell carcinoma, and high CXCL13 expression associated with a poor prognosis.^[23]^

A study found that docetaxel induced CD8+ T cell recruitment into the tumor microenvironment by enhancing the secretion of CXCL11, thus improving antitumor efficacy, and that increased CXCL11 expression was positively correlated with prolonged OS in lung cancer patients. This is also confirmed in our research. What is more, a high level of CXCL11 is associated with worse TNM staging in patients with pancreatic cancer (PC), enhancing the proliferation and metastasis of PC cells.^[24]^ Similarly, in our study, CXCL11 was found to express higher in the tissues of patients with HNC than in normal tissues, and was expressively connected with individual cancer staging in patients with HNC. In our study, through data analysis, we found that CXCL11 was significantly correlated with the pathological stage of patients with HNC (*P* ≤ .01). We can use the expression of CXCL11 to predict the disease process of patients with HNC, accurately judge the disease of patients, and assist clinicians to accurately judge the disease stage of patients with HNC. Thus, different treatment methods are formulated according to the disease process to improve the survival rate of patients.

Razise et al discovered that high expression of CXCL9 resulted in higher tumor-invasion lymphocyte density in tumors. High CXCL9 expression is a predictor of OS disadvantage.^[25]^ In our study, CXCL9 expression was strongly connected with clinical staging in patients with HNC and the overall survival time of patients with HNC (*P* ≤ .0038). CXCL9 can accurately diagnose patients with HNC in the above two aspects.

In TCGA database, the expression of CXCL10 in hepatocellular carcinoma tumor tissues was obviously higher than in non-tumor tissues.^[26]^ CXCL10, which is a ligand of CXCR3, is mainly secreted by monocytes, endothelial cells, fibroblasts, and cancer cells.^[27]^ The classic view on CXCL10 is that it prevents cancer through paracrine signaling as CXCL10 plays an important role in the recruitment and activation of immune cells.^[2]^ In our present study, it was found that CXCL10 was bounded up with the clinical stage of patients with HNC (*P* ≤ .01), and the expression level of CXCL10 was the highest in the CXCL chemokine family among HNC. In addition, elevated CXCL10 levels was obviously connected with lower OS (*P* ≤ .01)levels in patients with HNC, and these findings also increase the possibility that CXCL10 will become a prognostic marker for HNC. We can make bold guesses about CXCL9/10, which can play an important role in the future diagnosis of patients with HNC. We also plan to further verify CXCL9/10 by experiments in the follow-up research, so as to provide reliable serological indicators for clinicians. We also analyzed the gene correlation of CXCL8/9/10/11/13, and found that there was a significant correlation among other chemokines except the co-occurrence of CXCL8/13, especially the co-occurrence of *P*_(CXCL9__/10)_ ≤ .01, which showed that they were interdependent.

We analyzed the Gene ontology (GO) enrichment of CXCL8/9/10/11/13, in which Biological processes (BP) showed that it actually affected the development and prognosis of HNC through inflammatory reaction. In our research, we found that the position where CXCL8/9/10/11/13 performed its function was in cytoplasm. And through enrichment analysis of Molecular functions (MF), it is found that it has a relate influence on the occurrence and prognosis of HNC mainly by secreting related cytokines or promoting related cytokine activities, and through enrichment analysis of KEGG, it was found that the influence of CXCL8/9/10/11/13 on metabolic pathway can only play a positive correlation role through the interaction between cytokines and receptors.

We have made relevant statistical analysis on CXCL8/9/10/11/13 in infection immunity, autoimmunity and inflammatory response of related organs, and found that it has no significant statistical significance in HNC. However, we regret that there were fewer patients with HNC who meet the inclusion criteria, we have not collected enough patient samples for experimental verification, we will further verify them in immunohistochemistry, its gene and protein expression level.

This paper still has some limitations. At the first, all the relevant data analyzed in the study was collected through the online database, and there might be relevant heterogeneity between the data. Secondly, the scarcity of patients and the difficulty of obtaining pathological samples led to the defects of our experiment. We will collect the pathological samples of HNC patients in the next experiment, from the pathological point of view to the protein level, we tested it and confirmed our statistical analysis results.

## 5. Conclusion

In this study, our results suggest that chemokine CXCL8/9/10/11/13 may play a critical role in the development of HNC, and, according to relevant data, it may affect the survival and prognosis of patients with HNC. It may become a new biomarker for diagnosis, treatment and prognosis of HNC in the future.

Note 1 http://www.oncomine.org

Note 2 http://ualcan.path.uab.edu/

Note 3 http://gepia.cancer-pku.cn/

Note 4 http://kmplot.com/analysis/

Note 5 http://www.cbioportal.org

Note 6 http://www.string-db.org

Note 7 https://david.ncifcrf.gov/summary.jsp

## Author contributions

Zhenyu Zhao and Yuyu Ma: The writer of the paper.

Jie Lv; NaifeishaMaimaiti; Jingyi Zhang; madinaimu aibibula: Collection of data in the database.

Zhongcheng Gong and Bin Ling: The guidance of the paper.

Conceptualization: Zhongcheng Gong.

Data curation: madinaimu aibibula, Yuyu Ma.

Formal analysis: Zhenyu Zhao.

Methodology: Jie Lv.

Project administration: Bin Ling.

Resources: Hongyue Min, Naifeisha Maimaiti, Jingyi Zhang.
